# Supplementary figures and images for: Antioxidative Effects of Chrysoeriol via Activation of the Nrf2 Signaling Pathway and Modulation of Mitochondrial Function
Source: Molecules. 2021 Jan 9;26(2):313. doi: 10.3390/molecules26020313 (PMC7826659; doi:10.3390/molecules26020313)

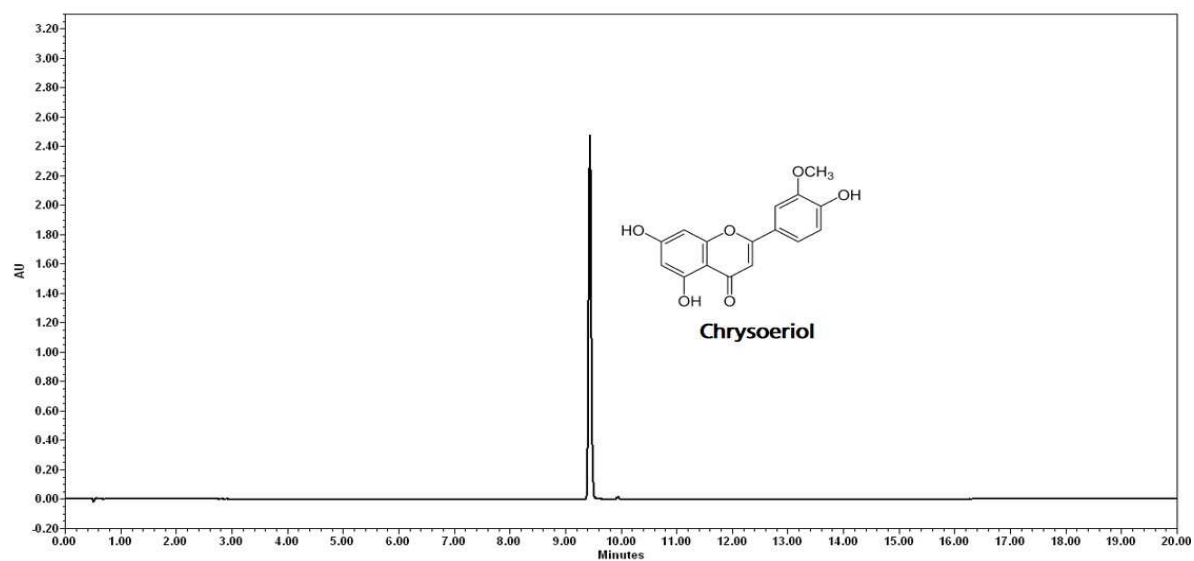

**Supplementary Figure 1. UHPLC chromatogram of chrysoeriol compound (purity > 98%)**

Supplement: Supplementary file 1 [file molecules-26-00313-s001.pdf]
